# Development of Assessment Tool to Measure Children's Knowledge on Dengue Prevention Activities in Malaysia

**DOI:** 10.1155/2022/2533900

**Published:** 2022-01-18

**Authors:** H. Siti Nur Farhana, J. Noorlaile, K. Manimaran, S. Kamarul Zaman, A. Normawati, Albeny Joslyn Panting

**Affiliations:** Institute for Health Behavioural Research, National Institute of Health, Ministry of Health Malaysia, Setia Alam, Selangor, Malaysia

## Abstract

Dengue is one of the public health concerns in developing and developed countries. Since the main control measure for dengue is health prevention and control activities, especially among children, it is essential to assess children's knowledge on dengue prevention activities in preventing the disease. This study aimed to develop an assessment tool (CAB-IHBR-Dengue-C-01) attempting to measure children's knowledge of dengue prevention activities in Malaysia. Twelve (12) pictorials with descriptions were developed to capture children's understanding of the correct method of dengue prevention activities. Cronbach' alpha test was used to assess the internal consistency of the items, and the test and retest reliability method was used to measure the consistency of the questionnaire. For test-retest reliability analysis, tests were conducted twice, with an interval of two (2) weeks from the first test. In total, 58 respondents comprising of Malay, Chinese, and Indian aged between 7 and 12 years old were included in this study. The results showed the intraclass correlation coefficient (ICC) score was 0.640; with moderate reliability; meanwhile, Cronbach's alpha score was 0.606. In conclusion, CAB-IHBR-Dengue-C-01 (Cognitive Affective Behaviour-Institute for Health Behavioural Research-Dengue-Children-Version 01) is reliable to be used.

## 1. Introduction

Dengue fever is a major public health concern, especially in tropical and subtropical areas [1, 2]. It was estimated by the World Health Organization (WHO) that around 50–100 million people get infected by dengue every year, globally. From this amount, 500,000 cases and 22,000 deaths were reported among children. More than two-thirds (70%) of these cases were reported in Asia [3].

Dengue fever is endemic in Malaysia, and it has become a major public health concern, with many outbreaks, particularly in urban areas [4]. It is a major health issue for the country and even supersedes other communicable diseases such as tuberculosis, HIV/AIDS, and malaria [5]. The number of dengue cases reported was staggering with 21,900 in 2012, and it has increased as much as twofold in just a year with 43,346 cases in 2013. Subsequently, the year 2015 recorded the highest number of dengue cases and dengue deaths with 120,836 and 336 cases, respectively [6].

The main strategy in preventing dengue is depending on vector control and preventing mosquito bites [7, 8]. In Malaysia, much of the potential breeding ground for Aedes came from unattended environments. These have contributed to the increasing number of dengue cases every year. Hence, primary prevention is widely regarded as the most effective strategy for preventing and controlling dengue fever. This includes using mosquito repellents, protective cloths, and destroying mosquito breeding sites by disposing stagnant water [9]. Nonetheless, the success depends on the level of public knowledge and practices about dengue as well as its prevention activities. Dengue fever can spread more easily if people are unaware of how it is transmitted and how to prevent it [10]. Moreover, previous study findings also emphasized the need to expand dengue fever preventive knowledge to control dengue outbreaks [11]. Knowledge and attitudes, knowledge and practice, and attitudes and practice all had a significant positive correlation. Furthermore, people with good knowledge were 2.7 times more likely to have positive attitudes, and people with positive attitudes were 2.2 times more likely to have good dengue practices [12].

The emphasis on knowledge of dengue prevention is very important since dengue can affect any age group. Children are at an equal risk of contracting dengue fever. Dengue fever affects an estimated 100 million individuals globally each year, with half a million cases of dengue haemorrhagic fever (DHF) fatalities in Asian countries. 90% of DHF patients are under the age of 15 [13]. It is essential to educate children from the beginning in order to develop people with good knowledge, attitude, and practice [14]. Studies have shown that individuals with higher knowledge of dengue tend to have a higher practice of prevention measures [15].

Based on this fact, there is a need to measure the level of dengue knowledge among children to effectively prevent and control dengue. Nevertheless, there are no standard tools available to measure the level of knowledge among children on dengue prevention activities. Prior studies have been using various sorts of tools for this purpose. Therefore, this study aims to develop and validate the tool for measuring children's knowledge of dengue prevention activities. This study hopes to provide a better understanding and act as a platform to improve children's knowledge in curbing dengue.

## 2. Methodology

### 2.1. Items Development

The instrument validation involved face validity and content validity which will be evaluated through pretesting (cognitive debriefing). For the reliability test, internal consistency (Cronbach alpha test) and test-retest were conducted. Items development was adapted from the National Health Morbidity Survey 2015 [16] and the Young Doctor Club Module under the dengue prevention submodule. This program was introduced by the Ministry of Health Malaysia in 1989 as a school-based health promotion program for primary school children to equip them with knowledge and skills to improve their health status [17].

**Figure 1:**
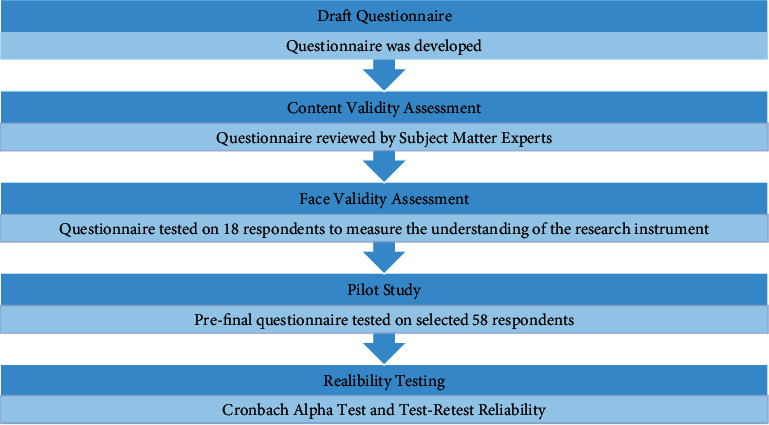
Flowchart of instrument validation.

The developed questionnaire, which was originally written in Malay, was sent to two independent translators for back-to-back translation (Malay-English-Malay) to assure the accuracy of the translation. The process of instrument validation is shown in Figure 1.

### 2.2. Content Validity Assessment

Content validity refers to the extent to which the instrument covers the content that it is meant to evaluate [18]. For content validity, the drafted questionnaire was reviewed by subject matter experts from Disease Control Division, Vector-Borne Disease Sector (Dengue Epidemiology Unit) Ministry of Health Malaysia and Health Education Division, and Ministry of Health Malaysia on the technical contents and to ensure the content of the instrument is fitting and in line with dengue prevention activities recommended by the Ministry of Health Malaysia. The items were amended and further refined based on the outcome of the discussion.

### 2.3. Face Validity Assessment

Face validity is the appropriateness, sensibility, or relevance of the test and its items as they appear to the persons answering the test and the degree to which test respondents regard the content of a test and its items as relevant to the context in which the test is administered [19]. The previous study indicates the number of respondents needed to test the survey prior to pilot testing or full-scale administration was at least 12–50 respondents [20]. In this study, face validity was conducted by a group of trained researchers on 18 children with similar characteristics to the actual respondents. Respondents were selected from the age range of 7–12 years old as the definition of a child from the WHO is a person who is 18 years old or younger [21]. Of the 18 respondents, 13 were from an urban area (Shah Alam) and another 5 of the respondents were from a rural area (Kapar). The average duration for the respondents to answer all the questions ranged from 10 to 15 minutes.

**Figure 2:**
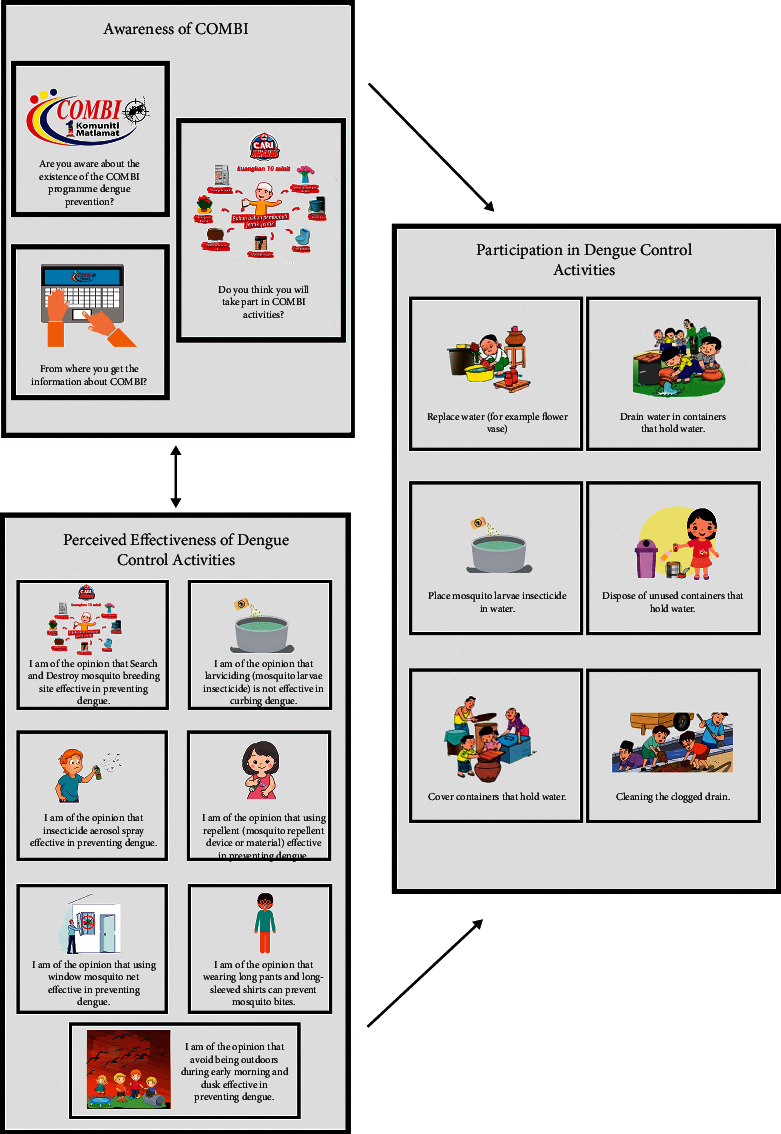
Proposed conceptual framework for CAB-IHBR-Dengue-C-01.

Initially, the questionnaire consisted of 3 sections, which were the awareness on Communication for Behavioural Impact (COMBI) that has been implemented in Malaysia to curb dengue, perceived effectiveness of dengue control activities (opinion-based), and participation in dengue control activities. The illustration of the framework is shown in Figure 2.

Awareness on COMBI is as follows:

1. Whether or not respondents are aware of COMBI program to curb dengue
2. Source of information (from where respondents get the information on COMBI)
3. Whether or not respondents have the intention to participate in COMBI activities

Perceived effectiveness of dengue control activities (opinion-based questions with 3 categories Likert scale: disagree, not sure, and agree):

1. Respondents' opinion on search and destroy Aedes mosquito breeding site effective in preventing dengue
2. Respondents' opinion on using mosquito larvae insecticide effective in preventing dengue
3. Respondents' opinion on using insecticide aerosol spray effective in preventing dengue
4. Respondents' opinion on using repellent (mosquito repellent device or material) effective in preventing dengue
5. Respondents' opinion on using window mosquito net effective in preventing dengue
6. Respondents' opinion on wearing long pants and long-sleeved shirts can prevent mosquito bites
7. Respondents' opinion on avoiding being outdoors during early morning and dusk effectively prevent dengue

Participation in dengue control activities (answer is Yes/No/not applicable option):

1. Replace water (for example, flower vase)
2. Drain water in containers that hold water
3. Place mosquito larvae insecticide in water
4. Dispose of unused containers that hold water
5. Cover containers that hold water
6. Cleaning the clogged drain

Based on the findings for the first section (awareness of COMBI), most respondents were unfamiliar with the terms Communication for Behavioural Impact (COMBI); hence, it is understandable for the respondents to not be able to answer the rest of the questions in that particular section. The decision to disregard this section was done as the questions were found to be irrelevant for this type of age group.

Finding for the second section showed the inability of the respondents to answer the questions due to the opinion-based questions are considered unsuitable for children due to their limited knowledge and experience on the matter, making it difficult for them to form an opinion. Besides, the items used to assess the perceived effectiveness of dengue prevention were removed considering the usual praxis; children are not allowed by their parents to administer chemicals such as repellent, aerosol, and other mosquito preventive chemicals, hence making it even harder for the respondents to form an opinion of the effectiveness.

As for the last section, the finding showed most of the target respondents answered based on their perception rather than their own practice. The removal of the items was done to increase direct understanding, relevant and clear questions for the children, hence, ensuring reliable data retrieval.

Further discussion was conducted with the subject matter experts, stakeholders, and National Health Morbidity Survey review panels on the outcome of the face validity. Suggestions and recommendations concerning the wordings and terminologies used were forwarded, and the instrument was revised to make it more appropriate, clear, and straightforward. Changes were made, including the removal of multiple choice options in the knowledge domain, rewording, and item rearrangement to improve readability and comprehensibility for the modified questionnaire.

As a final point, the decision was made to assess only on a single domain using a dichotomic type of question along with visual aids as a simple way to evaluate basic knowledge of dengue prevention activities specifically for children. This is in order to suit the objective of this instrument development to be used in the national survey with the need to produce a concise survey instrument. The questionnaire has been modified to 12 questions with visual aids.

### 2.4. Pilot Study

In this study, pilot testing has been conducted on 58 respondents, age ranging from 7 to 12 years old using the modified questionnaire followed by analyzing the reliability of the instrument. The duration for the respondents to answer all the questions took approximately 5–7 minutes compared to the previous questionnaire (15–20 minutes) as the questionnaire has been modified to be simpler for children to answer.

The modified questionnaire has been amended to one section with the emphasis on knowledge on dengue prevention activities. 12 pictorials with descriptions were developed to assist in capturing children's understanding of the correct method of dengue prevention activities. Respondents were required to write (✔**)** on the pictures showing the correct dengue prevention method and (✖) on the pictures showing the wrong dengue prevention method. The instrument measured the knowledge domain (Yes/No). Numerical score of 1 will be given to the correct answer and 0 to the incorrect answer. Possible scores ranged from 0 to 12. The overall number of items is 12. The items in the questionnaire are as follows:

1. Cover containers that hold water
2. Drain water in containers that hold water
3. Replace water (for example, flower vase)
4. Use repellent (mosquito repellent device or material)
5. Littering
6. Wearing clothing that could expose to mosquito bites
7. Place mosquito larvae insecticide in water
8. Cleaning the clogged drain
9. Playing during dusk
10. Communal work (gotong-royong) activity
11. Dumping rubbish into the river
12. Using insecticide aerosol spray

Cronbach' alpha test and a test-retest analysis were used to assess the instrument's reliability. For this survey, Cronbach' alpha test was used to assess the internal consistency of the items [22], while the test-retest reliability approach was used to assess the instrument's stability across time [23]. The instrument's stability refers to how similar the results are on two separate occasions or during a test-retest process [24]. For Cronbach' alpha test, the test was performed based on the pilot test's result from 58 respondents, and for test-retest reliability analysis, tests were conducted twice on 10 respondents with an interval of two weeks from the first test.

## 3. Results

A total of 58 respondents had participated in this pilot test; 36 (62.1%) respondents were from urban areas (Putrajaya, Shah Alam, Setia Alam, Subang Jaya, and Kuching) and 22 (37.9%) respondents were from rural areas (Hulu Selangor and Meru, and Selangor). The details on the respondents' characteristics are given in Table 1.

### 3.1. Reliability Testing

Cronbach's alpha value for the original and the revised tool is given in Table 2. The value of Cronbach's alpha for all the initial 12 items was 0.552. Items B12, B6, and B7 were removed from the questionnaire to increase Cronbach's alpha value to 0.606.

**Figure 3:**
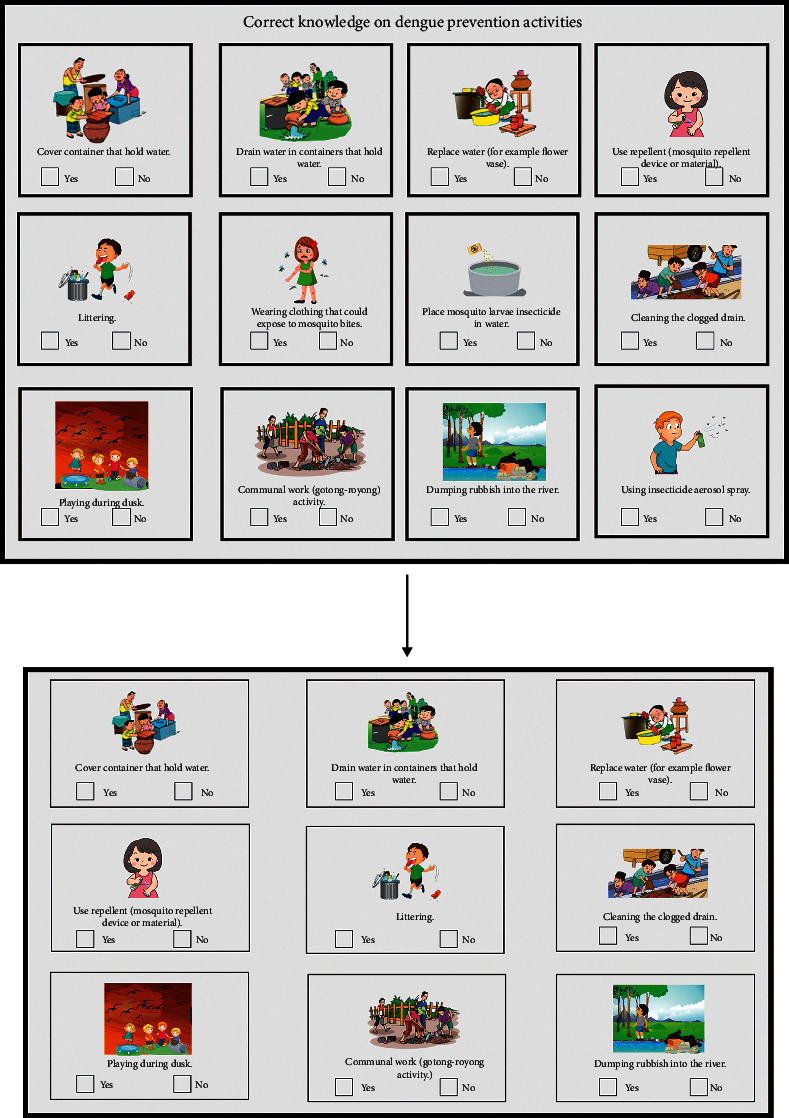
Correct knowledge on Dengue prevention activities.

The questionnaire is shown in Figure 3.

### 3.2. Test and Retest Reliability

The test and retest reliability method was used to measure the consistency of the questionnaire. For test-retest reliability analysis, tests were conducted twice, with an interval of two weeks from the first test. The data were collected from 10 children attempting to measure the consistency of the result.  Table 3 presents the result of interclass correlation for the tool based on single measures. The ICC value for the final tool was 0.640.

## 4. Discussion

Knowledge of school children plays a vital role in implementing remedial actions for dengue eradication. Despite such importance of children's knowledge for dengue prevention, less research attention has been paid by the researchers to examine children's knowledge of dengue prevention [25]. There is an immense need to develop an instrument for measuring children's knowledge on dengue prevention control, especially when most children all over Malaysia have been exposed to the knowledge regarding dengue prevention control in the Kelab Doktor Muda (Young Doctor Club) module. Kelab Doktor Muda was established by the Ministry of Health Malaysia with the aim to train a group of schoolchildren as peer educators in assisting their peers in adopting healthy practices [26]. As of 2019, Kelab Doktor Muda has been implemented in 3286 schools nationwide and has reached 1,388,790 children [27]. Therefore, to determine to which extend children understand the correct way of preventing dengue, a national health morbidity survey will be conducted, and this instrument was developed for this national study. The Ministry of Health Malaysia finds it is very important to measure the children's level of knowledge on proper dengue prevention as introducing knowledge of dengue prevention at an earlier age can be a basis for adult health behaviours [28]. It is of the utmost importance to curb dengue since the incidence of dengue infection is predicted to become nearly four times higher in 2020 than it was in 2010 and nearly six times higher in 2040 than it was in 2010 [29].

This study developed a nine-item assessment tool to measure children's knowledge on dengue prevention activities. Each item used a dichotomous questionnaire of “Yes” and “No” as an answer as well as visual aids. The study carried out has a sample size of 58 participants. As this is a pilot study, researchers followed the principles of pilot test participants, and the sample size is recommended to be larger than 30 and less than 500 participants [30]. Furthermore, researchers have also selected over 50 participants, in order to have at least 5 participants per question in the survey. While developing the tool, this study also assessed the validity and reliability to ensure the tool is appropriate. Results of this study indicated that the tool has acceptable validity and reliability.

Based on the content review from the subject matter experts, the final tool is in concordance with dengue prevention activities suggested by the Ministry of Health Malaysia. However, rather than assessing initial awareness, perceived effectiveness, and participation in dengue prevention activities, the final tool was modified only to include the knowledge domain on the prevention of dengue. This was due to the initial questionnaire being above the cognitive level for children aged 7–12 years old, eventhough it was a researcher-guided questionnaire. According to Fuchs, children' age has a limited cognitive function for the complex questionnaire. Eventhough it is believed that development does not follow a strict age-related logic for all people, it is clear that the cognitive abilities of 10-year-old children and 17-year-old adolescents vary significantly. It is debatable whether young children are capable of simultaneously remembering all of the information provided by a survey item while processing each question and the questionnaire as a whole. It is also unclear how they process and link the relevant information provided contrastingly to older participants [31]. Due to this limitation, visual aids were added to the questionnaire to assist the children in improving their understanding of each item. Visual aids such as flashcards are frequently used to build automaticity and improve reading fluency [32].

Cronbach's alpha (*α*) internal consistency reliability in this study reached the acceptable threshold value. In social science, the acceptable *α* value is 0.60 [33], which is also practiced by other researchers. Intraclass correlation coefficient (ICC) was used as reliability index in test and retest reliability, and according to Koo and Li, the ICC score for the values less than 0.5 indicate poor reliability, while values between 0.5 and 0.75 indicate moderate reliability, 0.75 and 0.9 indicate good reliability, and values greater than 0.90 indicate excellent reliability [34]. The ICC score for this instrument was 0.640; hence, the score indicated moderate reliability.

## 5. Limitation

The tool may be used as a simple guide to aid researchers in studies related to children's knowledge on dengue prevention activities, and a more in-depth instrument can be developed to further improve this instrument. While the sample size for the study is enough, the additional sample size may add further value to the validation test.

## 6. Conclusion

This study indicated that CAB-IHBR-Dengue-C-01 was reliable to be used to measure children's knowledge on dengue prevention activities. In Malaysia, there is a limited study conducted on children in regard to dengue prevention activity probably due to no appropriate instrument that can be used in assessing the knowledge. Further improvement is needed to develop an appropriate instrument to measure the attitude and practice of children in regard to dengue prevention activities. This study hopes to contribute to facilitate other research studies that aim to assess children' knowledge on dengue prevention.

## Figures and Tables

**Table 1:** Characteristics of the respondents (*n* = 58).

Characteristics	Frequency	Percentage (%)
Area		
Urban	36	62.1
Rural	22	37.9
Gender		
Male	29	50.0
Female	29	50.0
Race		
Malay	37	63.8
Chinese	3	5.2
Indian	16	27.6
Bumiputera Sabah	2	3.4

**Table 2:** Cronbach's alpha value for original and revised tool (*n* = 58).

	Number of items	Item dropped	Cronbach's alpha
Original	12	—	0.552
First revision	11	B12	0.587
Second revision	10	B12, B6	0.594
Third revision (final tool)	9	B12, B6, B7	0.606
Items			
B1. Cover containers that hold water			Retained
B2. Drain water in containers that hold water			Retained
B3. Replace water (for example, flower vase)			Retained
B4. Use repellent (mosquito repellent device or material)			Retained
B5. Littering			Retained
B6. Wearing clothing that could expose to mosquito bites			Deleted
B7. Place mosquito larvae insecticide in water			Deleted
B8. Cleaning the clogged drain			Retained
B9. Playing during dusk			Retained
B10. Communal work (gotong-royong) activity			Retained
B11. Dumping rubbish into the river			Retained
B12. Using insecticide aerosol spray			Deleted

**Table 3:** Interclass correlation of the final tool.

	Intraclass correlation
Single measures	0.640

## Data Availability

The data are not part of an online database but can be requested by writing to the Director of the Institute for Health Behavioural Research, National Institutes of Health, Ministry of Health Malaysia.
